# Loneliness, quality of life, and life satisfaction among older adults: the mediating role of resilience

**DOI:** 10.3389/fpubh.2026.1947235

**Published:** 2026-09-29

**Authors:** Jae Hee Kim

**Affiliations:** Department of Nursing, Daejin University, Pocheon-si, Gyeonggi-do, Republic of Korea

**Keywords:** Korea, life satisfaction, loneliness, mediation, older adults, quality of life, resilience

## Abstract

Loneliness is a psychosocial factor associated with poorer quality of life and subjective wellbeing in later life. Few studies have examined whether resilience helps explain these associations among Korean older adults. This cross-sectional study examined whether resilience statistically mediated the associations of loneliness with quality of life and life satisfaction among Korean older adults. Data were drawn from the National Mental Health Survey of Korea 2021. Of 1,037 eligible adults aged 65–79 years, 1,018 participants with complete data were included in the analyses. Loneliness-related social disconnectedness, resilience, quality of life, and life satisfaction were assessed using survey-based measures, and mediation was examined using covariate-adjusted regression models and Sobel tests. Loneliness was negatively correlated with resilience (*r* = −0.42), quality of life (*r* = −0.41), and life satisfaction (*r* = −0.42), whereas resilience was positively correlated with quality of life (*r* = 0.34) and life satisfaction (*r* = 0.41). Mediation analyses showed patterns consistent with partial mediation by resilience in the association between loneliness and quality of life (Sobel *Z* = −5.19, *p* < 0.001) and in the association between loneliness and life satisfaction (Sobel *Z* = −6.96, *p* < 0.001). The corresponding final models had adjusted R^2^ values of 0.196 and 0.249, respectively. By examining both positive wellbeing outcomes, the study extends prior analyses of these national data beyond psychiatric outcomes and highlights resilience as a potentially modifiable psychological resource. Because the data were cross-sectional and key constructs were measured using brief survey indicators, causal interpretations should be avoided. Further longitudinal and intervention research should test whether resilience-building approaches, combined with efforts to reduce loneliness and strengthen social connection, can improve wellbeing in later life.

## 1 Introduction

Korean society is aging rapidly, raising important psychological and public health questions about how to maintain and promote mental health and quality of life in later life. Statistics Korea projects that the proportion of adults aged 65 years or older will increase from 19.2% in 2024 to 47.7% in 2072 (1). This demographic shift involves more than longer life expectancy. It is accompanied by changes in social relationships, health vulnerability, living alone, weakened family support, and unequal opportunities for community participation. Among these issues, loneliness has emerged as a salient socioemotional experience that can undermine psychological wellbeing in later life and is closely related to subjective distress, physical health and social functioning (2). The WHO Commission on Social Connection identified loneliness as an important social determinant of health and wellbeing across the life course (3). The National Academies of Sciences, Engineering, and Medicine (4) also characterized loneliness among older adults as a public health concern that health care systems should screen for and address.

Quality of life and life satisfaction are widely used outcome indicators for evaluating wellbeing in later life (5, 6). Quality of life refers to a broad appraisal of life that includes physical, psychological, social, and environmental domains, whereas life satisfaction focuses on how individuals cognitively evaluate their lives as a whole (7). Examining both outcomes is therefore useful for understanding how loneliness is related to wellbeing in older adults. Quality of life can reflect health and daily functioning, while life satisfaction more directly captures subjective appraisal. Considering both outcomes allows a broader interpretation of the psychological relevance of loneliness.

Resilience, defined as the capacity to adapt and recover when facing stress, adversity, and negative life events, may be considered an important psychological resource in the process through which loneliness is associated with quality of life and life satisfaction. Systematic reviews and meta-analyses on resilience in later life have reported associations between resilience and subjective health, mental health, life satisfaction, and successful aging (8, 9). Thus, even when older adults experience loneliness, resilience-related resources such as stress coping, emotion regulation, self-efficacy, and meaning-making may help explain why the association between loneliness and diminished wellbeing varies across individuals.

Studies of loneliness, resilience, mental health, and quality of life suggest that resilience may play a mediating role in associations between loneliness and wellbeing. Gerino et al. (10) reported that resilience helped explain the association between loneliness and quality of life among older adults. Wen et al. (11) also found that resilience may mediate the association between loneliness and depression in older adults. More recently, Kushta and Moliner Albero (12) reported a mediating role of resilience in the association between loneliness and quality of life among institutionalized older adults. However, much of the existing evidence has been based on specific regions, institutionalized older adults, older women living alone, convenience samples, or negative mental health outcomes such as depression. Studies using nationwide data to examine loneliness, resilience, quality of life, and life satisfaction together among older adults remain limited.

Korean studies have also examined loneliness and social isolation in relation to mental health outcomes, but the available evidence remains concentrated on negative outcomes or specific subgroups. Lim et al. (13) examined community-dwelling older women living alone in South Korea and found that subjective physical health, resilience, and social support mediated the association between loneliness and depression. Recent studies using Korean national data have further shown that loneliness and social isolation may be associated with major mental disorders and other mental health problems, including depressive disorders, anxiety disorders, and suicidal behavior (14, 15). These studies provide important evidence on the mental health risks associated with loneliness and social isolation in Korea, but they do not directly address positive wellbeing outcomes in later life. Research on mental health in later life should also consider quality of life and life satisfaction, not only the presence or absence of mental disorders. Resilience has theoretical and practical significance because it is a psychological resource that may be developed through intervention and may therefore be relevant to improving wellbeing among older adults who experience loneliness (16, 17).

Accordingly, this study used data from the National Mental Health Survey of Korea 2021 (18), a nationwide survey of Korean adults, to examine associations among loneliness, resilience, quality of life, and life satisfaction in Korean older adults. Specifically, the study first analyzed the associations among loneliness, resilience, quality of life, and life satisfaction. It then examined whether resilience statistically mediated the associations between loneliness and quality of life and between loneliness and life satisfaction. Through these analyses, this study moves beyond a direct-association model of loneliness and wellbeing in later life and examines resilience as a potentially modifiable psychological resource within these associations.

## 2 Materials and methods

### 2.1 Data source and participants

#### 2.1.1 Data source

This study was a secondary analysis of data from the National Mental Health Survey of Korea (NMHSK), a national survey dataset. The NMHSK has been conducted every 5 years since 2001 by the Ministry of Health and Welfare and the National Center for Mental Health. It is based on the Korean version of the Composite International Diagnostic Interview (K-CIDI). The survey uses stratified cluster sampling to select sample households, from which one adult aged 18–79 years per household is interviewed. The survey covers mental disorders excluding schizophrenia, mental health service utilization, perceptions of mental disorders, and mental health indicators including quality of life. The 2021 NMHSK used in this study was conducted from June to August 2021.

#### 2.1.2 Participants

The source sample included 1,037 adults aged 65–79 years. Responses from 90 participants (1.8%) were incomplete and were excluded from the analyses. The final analytic sample therefore comprised 1,018 participants. This sample size exceeded the minimum required sample size of 114 calculated using G*Power 3.0.10 for regression analysis, based on an effect size of 0.15, a two-tailed significance level of 0.05, statistical power of 0.80, and nine predictors including expected covariates and independent variables.

### 2.2 Study variables

#### 2.2.1 Sociodemographic characteristics

Sociodemographic characteristics included sex, age, educational attainment, type of health insurance, and living with family members. Age was categorized as 65–69, 70–74, or 75–79 years. Educational attainment was categorized as elementary school or less, middle or high school, or college or higher. Type of health insurance was classified as either National Health Insurance (NHI) or Medical Aid, and was used as an indirect proxy for household economic status. For living with family members, cases recorded as “zero,” indicating no currently cohabiting family members in the original data, were recoded as “none.”

#### 2.2.2 Loneliness

Loneliness was measured using the Loneliness and Social Isolation Scale developed by Hwang et al. (19) to assess loneliness and social disconnectedness among outpatients and caregivers at a tertiary hospital. The instrument comprises six items across three domains: loneliness (2 items), social support (2 items), and social networks (2 items). At the time of development, Cronbach’s alpha for internal consistency was 0.77. In this study, Cronbach’s alpha was 0.67, indicating modest internal consistency. Findings using this variable should therefore be interpreted with caution.

#### 2.2.3 Quality of life

Quality of life was assessed with a single item asking, “How would you rate your quality of life?” Responses were recorded on a 5-point scale ranging from “very poor” (1) to “very good” (5).

#### 2.2.4 Life satisfaction

In the original data, life satisfaction was assessed with a single item asking, “Considering your life overall, how satisfied are you with your current life?” Responses were recorded on an 11-point scale. Because the response distribution showed a pronounced central tendency and very low proportions at both extremes (0–2 and 8–10), the scores were recategorized into a 5-point scale to improve the stability of estimates for extreme categories: scores of 0–2 were re-coded as 1 (very dissatisfied), 3–4 as 2 (dissatisfied), 5 remained as 3 (neutral), 6–7 as 4 (satisfied), and 8–10 as 5 (very satisfied).

#### 2.2.5 Resilience

Resilience was measured using 10 items from the 25-item scale developed by Connor and Davidson (20), as used in the NMHSK. The specific items were not disclosed in the mental health survey due to copyright issues. However, in the original development study, the 25-item scale was described as covering adaptability, problem-solving ability, overcoming stress, and achievement. Each item is rated on a 5-point scale ranging from “not true at all (0)” to “true nearly all the time (4)“, with higher scores indicating greater resilience. Cronbach’s alpha was 0.93 in the original development study and 0.94 in this study.

### 2.3 Data analysis

All statistical analyses were performed using IBM SPSS Statistics for Windows, version 31.0.

Differences in resilience, quality of life, and life satisfaction by participants’ sociodemographic characteristics were examined using independent-samples *t*-tests or analysis of variance (ANOVA). Associations among loneliness, resilience, quality of life, and life satisfaction were examined using Pearson’s correlation coefficients.

The mediating role of resilience in the associations between loneliness and quality of life and between loneliness and life satisfaction was examined using the causal-steps approach proposed by Baron and Kenny (21) through a series of three regression steps. In Step 1, the association between the independent variable, loneliness, and the mediator, resilience, was analyzed. In Step 2, the association between loneliness and each dependent variable, quality of life or life satisfaction, was analyzed. In Step 3, loneliness and resilience were entered simultaneously to predict the dependent variable. The Sobel test was used to evaluate the statistical significance of the indirect effect. Because the study used cross-sectional data, the mediation analyses were interpreted as statistical mediation patterns rather than causal mediation.

### 2.4 Ethical consideration

This study was approved in advance by D. University Institutional Review Board (1040656-202606-HR-01-12).

## 3 Results

### 3.1 Differences in resilience, quality of life, and life satisfaction by sociodemographic characteristics

Participants’ resilience differed significantly by sex (*t* = −4.32, *p* < 0.001), age group (*F* = 4.28, *p* = 0.014), educational attainment (*F* = 13.47, *p* < 0.001), type of health insurance (*t* = 2.06, *p* = 0.039), and living with family members (*t* = 2.73, *p* = 0.006). Resilience scores were higher among women than men, those with NHI rather than Medical Aid, and those living with family members. Resilience also differed by age group and educational attainment.

Quality of life differed significantly by sex (*t* = −2.92, *p* = 0.004), educational attainment (*F* = 7.11, *p* < 0.001), type of health insurance (*t* = 5.39, *p* < 0.001), and living with family members (*t* = 4.70, *p* < 0.001). Quality of life scores were higher among women, those with NHI rather than Medical Aid, and those living with family members; scores also differed by educational attainment.

Life satisfaction differed significantly by sex (*t* = −4.02, *p* < 0.001), educational attainment (*F* = 6.45, *p* = 0.002), type of health insurance (*t* = 6.68, *p* < 0.001), and living with family members (*t* = 4.89, *p* < 0.001). Life satisfaction scores were higher among women, those with NHI rather than Medical Aid, and those living with family members; scores also differed by educational attainment (Table 1).

**Table 1:** Resilience, quality of life, and life satisfaction by sociodemographic characteristics (*N* = 1,037).

Variable	Category(n)	Resilience		Quality of life		Life satisfaction	
		M ± SD	*t(p) or F(p)*	M ± SD	*t(p) or F(p)*	M ± SD	*t(p) or F(p)*
Sex	Male (528)	22.10 ± 7.18	−4.32 (*p* < 0.001)	3.24 ± 0.71	−2.92 (*p* = 0.004)	3.73 ± 0.94	−4.02 (p < 0.001)
	Female (509)	24.00 ± 6.92		3.37 ± 0.67		3.96 ± 0.86	
Age(yr)	65–69 (574)	23.61 ± 6.82	4.28 (0.014)	3.33 ± 0.68	0.98 (0.375)	3.89 ± 0.88	1.79 (0.167)
	70–74 (282)	22.41 ± 7.36		3.26 ± 0.73		3.79 ± 0.91	
	75–79 (181)	22.18 ± 7.52		3.29 ± 0.67		3.78 ± 0.99	
Education	Elementary school or less (287)	22.03 ± 7.15	13.47 (< 0.001)	3.22 ± 0.71	7.11 (< 0.001)	3.77 ± 0.94	6.45 (0.002)
	Middle or high school (692)	23.09 ± 6.97		3.31 ± 0.68		3.84 ± 0.90	
	College or higher (58)	27.28 ± 7.17		3.59 ± 0.59		4.24 ± 0.82	
Health insurance	NHI (981)	23.25 ± 6.97	2.06 (0.039)	3.34 ± 0.67	5.39 (< 0.001)	3.90 ± 0.87	6.68 (< 0.001)
	Medical Aid (37)	20.84 ± 7.56		2.73 ± 0.73		2.92 ± 1.09	
Living with family members	Yes	23.38 ± 7.01	2.73 (0.006)	3.36 ± 0.67	4.70 (<0.001)	3.92 ± 0.87	4.89 (<0.001)
	No	21.96 ± 7.33		3.12 ± 0.73		3.60 ± 0.99	

### 3.2 Correlations among variables

Loneliness was negatively correlated with resilience (*r* = −0.42, *p* < 0.001), quality of life (*r* = −0.41, *p* < 0.001), and life satisfaction (*r* = −0.42, *p* < 0.001). Resilience was positively correlated with quality of life (*r* = 0.34, *p* < 0.001) and life satisfaction (*r* = 0.41, *p* < 0.001). Quality of life was positively correlated with life satisfaction (*r* = 0.70, *p* < 0.001) (Table 2).

**Table 2:** Pearson correlations among study variables.

Variables	Loneliness	Resilience	Quality of life	Life satisfaction
Loneliness	1	−0.42 (< 0.001)	−0.41 (< 0.001)	−0.42 (<0.001)
Resilience		1	0.34 (< 0.001)	0.41 (<0.001)
Quality of life			1	0.70 (<0.001)

### 3.3 Statistical mediation by resilience

Multicollinearity and autocorrelation were not evident in the regression models. Variance inflation factor (VIF) values ranged from 1.05 to 4.80, below the conventional threshold of 10. Durbin-Watson statistics ranged from 1.89 to 1.92, approximating 2.00.

The results were consistent with a partial mediating role of resilience in the association between loneliness and quality of life. In Step 1, loneliness was significantly associated with resilience (*β* = −0.38, *t* = −12.91, *p* < 0.001). In Step 2, loneliness was significantly associated with quality of life (*β* = −0.36, *t* = −12.13, *p* < 0.001). In Step 3, resilience was significantly associated with quality of life (*β* = 0.17, *t* = 5.56, *p* < 0.001), and the model explained 19.6% of the variance. The association between loneliness and quality of life was attenuated in Step 3 relative to Step 2. Because loneliness remained significantly associated with quality of life in Step 3, the mediation pattern was partial. The Sobel test supported the statistical significance of the indirect effect (*Z* = −5.19, *p* < 0.001) (Table 3 and Figure 1).

**Table 3:** Mediating effect of resilience on the relationship between loneliness and quality of life.

Variables	*β*	*t(p)*	*R* ^2^	Adjusted *R*^2^	*F(p)*
Step 1. Loneliness → Resilience^a^	−0.38	−12.91 (<0.001)	0.185	0.179	28.55 (<0.001)
Step 2. Loneliness → Quality of life^b^	−0.36	−12.13 (<0.001)	0.177	0.172	36.07 (<0.001)
Step 3. Loneliness → Quality of life^b^	−0.30	−9.33 (<0.001)	0.201	0.196	36.26 (<0.001)
Resilience → Quality of life	0.17	5.56 (< 0.001)			
Sobel test: *Z* = −5.19 (< 0.001)					

^a^Adjusted for sex, age, education, health insurance, and living with family members. ^b^Adjusted for sex, education, health insurance, and living with family members.

**Figure 1:**
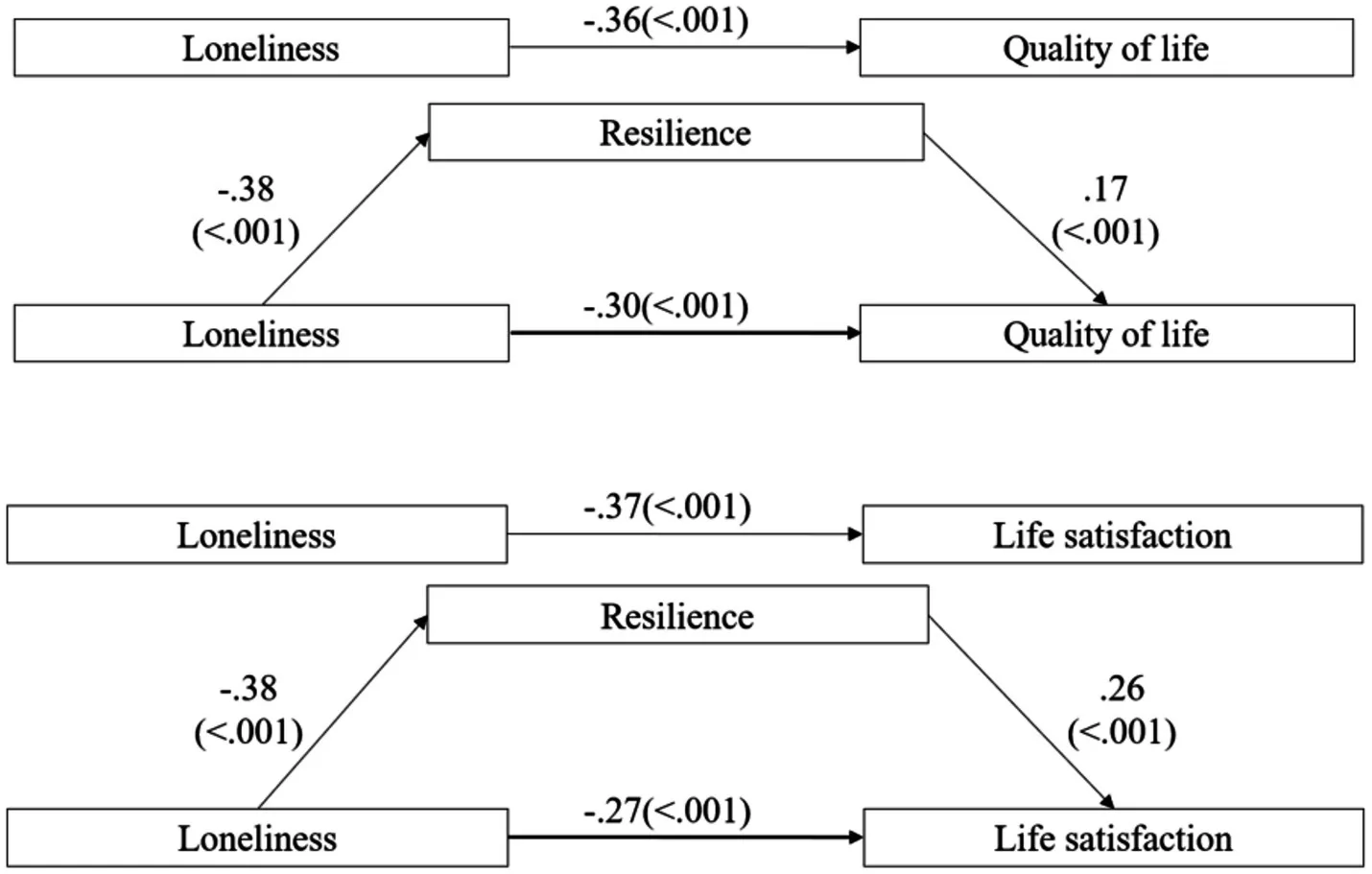
Proposed statistical mediation models linking loneliness, resilience, and wellbeing outcomes.

The results were also consistent with a partial mediating role of resilience in the association between loneliness and life satisfaction. In Step 1, loneliness was significantly associated with resilience (*β* = −0.38, *t* = −12.91, *p* < 0.001). In Step 2, loneliness was significantly associated with life satisfaction (*β* = −0.37, *t* = −12.50, *p* < 0.001). In Step 3, resilience was significantly associated with life satisfaction (*β* = 0.26, *t* = 8.66, *p* < 0.001), and the model explained 24.9% of the variance. The association between loneliness and life satisfaction was attenuated in Step 3 relative to Step 2. Because loneliness remained significantly associated with life satisfaction in Step 3, the mediation pattern was partial. The Sobel test supported the statistical significance of the indirect effect (*Z* = −6.96, *p* < 0.001) (Table 4 and Figure 1).

**Table 4:** Statistical mediation analysis of the association between loneliness and life satisfaction.

Variables	*β*	*t(p)*	R^2^	Adjusted R^2^	*F(p)*
Step 1. Loneliness → Resilience^a^	−0.38	−12.91 (< 0.001)	0.185	0.179	28.55 (< 0.001)
Step 2. Loneliness → Life satisfaction^b^	−0.37	−12.50 (< 0.001)	0.199	0.194	41.69 (< 0.001)
Step 3. Loneliness → Life satisfaction^b^	−0.27	−8.78 (< 0.001)	0.254	0.249	49.06 (< 0.001)
Resilience → Life satisfaction	0.26	8.66 (< 0.001)			
Sobel test: *Z* = −6.96 (< 0.001)					

^a^Adjusted for sex, age, education, health insurance, and living with family members. ^b^Adjusted for sex, education, health insurance, and living with family members.

## 4 Discussion

This study examined associations among loneliness, resilience, quality of life, and life satisfaction in Korean older adults using data from the NMHSK 2021. It also tested whether resilience statistically mediated the associations between loneliness and two wellbeing indicators. Three main findings emerged. First, loneliness was negatively associated with resilience, quality of life, and life satisfaction. Second, resilience was positively associated with quality of life and life satisfaction. Third, the results were consistent with partial mediation by resilience in the associations between loneliness and quality of life, and between loneliness and life satisfaction. These findings suggest that loneliness in later life is related not only to lower quality of life and lower life satisfaction, but also to lower levels of an intrapersonal psychological resource, in this case resilience, which may partly account for these associations. Because the study was cross-sectional, the pathways discussed below should be understood as theoretically plausible interpretations of statistical mediation patterns rather than established causal relationships.

The findings are broadly consistent with previous literature identifying loneliness as an important psychosocial risk factor for health and wellbeing in later life. Prior studies have reported that loneliness and social isolation may be associated with mental health, physical health, and mortality risk (3, 4, 22). The WHO Commission on Social Connection framed loneliness and social isolation as global health and wellbeing concerns, emphasizing that loneliness should not be viewed as a mere personal emotion but as a psychosocial factor linking social connection to health. This study extends that international discussion to positive wellbeing indicators, namely quality of life and life satisfaction, among Korean older adults.

The study also contributes to recent Korean research. Studies using the 2021 NMHSK have examined loneliness and social isolation in relation to depressive disorders, anxiety disorders, alcohol use disorder, and suicidal behavior (14, 15). These studies suggest that loneliness, particularly when accompanied by social isolation, is associated with elevated mental health risk, but they do not directly address positive wellbeing in later life. By focusing on quality of life and life satisfaction in an older-adult subsample drawn from the same nationwide survey, this study extends loneliness research from pathology-oriented outcomes to wellbeing-oriented outcomes.

The mediating role of resilience may be interpreted as a psychological pathway linking loneliness and wellbeing in later life. Previous studies focusing on quality of life provide the closest basis for this interpretation. Gerino et al. (10) reported that loneliness, resilience, mental health, and quality of life were structurally interconnected among older adults and that resilience and mental health mediated the association between loneliness and mental and physical quality of life. Kushta and Moliner Albero (12) also reported that resilience mediated the association between loneliness and quality of life among institutionalized older adults. These studies suggest that resilience may help explain the link between loneliness and quality of life, but they were conducted in specific national, cultural, or institutional contexts and did not examine life satisfaction together with quality of life. Evidence from depression-focused research points in a related direction. Wen et al. (11), using meta-analytic structural equation modeling, suggested that the association between loneliness and depression in older adults could be partly explained by resilience and social support. Systematic reviews and meta-analyses have also reported associations of resilience with subjective health, mental health, life satisfaction, and successful aging in later life (8, 9), supporting the interpretation of resilience as a key psychological resource in wellbeing. The present study extends this literature by using nationwide Korean mental health survey data and by examining both quality of life and life satisfaction as positive wellbeing indicators. The findings indicate that the partial mediation pattern involving loneliness and resilience was observed not only for quality of life, which has been examined in prior studies, but also for life satisfaction among Korean older adults.

A common starting point in both mediation models was the negative association between loneliness and resilience. This association can be interpreted in terms of emotional and social resources. First, loneliness may be accompanied by negative emotional states such as stress and depressed mood, which may undermine emotional resources and coping capacity (23, 24). Also, because resilience is itself an emotional and coping resource, it may be lower among those experiencing loneliness. Second, loneliness may weaken resilience by reducing the social resources that support it. For example, an event that precipitates loneliness may also reduce contact with others, emotional support, or other forms of social resources. From this perspective, the observed association may reflect not only an independent association of loneliness with resilience but also the influence of antecedent conditions that shape both loneliness and resilience.

The finding that resilience partially mediated the association between loneliness and quality of life can be interpreted in several ways. To begin with, loneliness may be negatively related to quality of life because loneliness itself can be appraised as an adverse life condition, especially in the psychological domain. Loneliness may also worsen other life conditions, including physical health, psychological state, daily functioning, and social role performance, which may then be reflected in quality of life. In addition, events that lead to loneliness, such as social exclusion or loss of family members or close associates, may also directly worsen life circumstances. In such cases, a common antecedent may shape both loneliness and quality of life, making the association between the two appear stronger.

The positive association between resilience and quality of life may be explained as follows. First, older adults with higher resilience may evaluate the same adverse life conditions less negatively because they perceive themselves as able to cope with and overcome difficulties. Resilience may also be related to quality of life because earlier use of resilience may have helped individuals improve the objective conditions of life. In this case, the positive evaluation of quality of life may reflect not only cognition but also past efforts to change the conditions being evaluated. Finally, resilience itself may be appraised as part of one’s life conditions, particularly as a psychological resource that is either already present or actively developed through experience.

The mediation by resilience in the association between loneliness and quality of life was partial. This indicates that loneliness was still associated with quality of life after resilience was included in the model. Some pathways may therefore connect loneliness to quality of life without passing through resilience. For example, if loneliness itself is perceived as an adverse life condition, resilience is not required to explain its association with quality of life. Similarly, if an event that produced loneliness is itself an adverse life condition, that event may be related to quality of life independently of resilience. It is also possible that loneliness leaves a lasting imprint on one’s appraisal of life, even when later coping resources improve.

Another major finding is that resilience also partially mediated the association between loneliness and life satisfaction. Loneliness may be negatively related to life satisfaction because it can function as an evaluative criterion—that is, a life marked by loneliness may be appraised as less satisfying. This explanation is supported by the fact that loneliness is generally perceived as a negative emotion or experience, and that social connection is central to human wellbeing. Social comparison may further intensify this association if individuals compare their own loneliness with others’ apparent connectedness. Loneliness may also worsen other life conditions, which can then be directly or indirectly related to lower life satisfaction. Finally, loneliness may be related to factors closely tied to life satisfaction, such as self-worth, meaning in life, perceived control, and anxiety. If loneliness is interpreted as a failure to form or maintain social connections, this interpretation may undermine self-worth, particularly when loneliness persists.

Resilience was positively associated with life satisfaction. Because both constructs include subjective and cognitive elements, the association may be closer than the association between resilience and quality of life. Resilience itself may become part of how individuals evaluate life satisfaction, especially when resilience is socially valued and internalized as a desirable attribute. Resilience may also be related to life satisfaction through other psychological resources such as self-esteem and self-efficacy. For example, perceiving oneself as mentally healthy and capable of coping with adverse situations may support satisfaction with life. Past experiences of overcoming, or failing to overcome, adversity through resilience may also contribute to life satisfaction.

The mediation by resilience in the association between loneliness and life satisfaction was also partial. This suggests that loneliness may be related to life satisfaction directly or through third variables not included in the present model. For one thing, loneliness itself may serve as a criterion for evaluating life satisfaction. In addition, the experience of loneliness at some point in life may have lasting relevance for how individuals evaluate their lives, regardless of whether it is later reduced or overcome.

This study distinguished quality of life from life satisfaction as two dependent variables. Although the two constructs overlap, they are not identical. Quality of life reflects evaluations of objective and subjective life conditions, whereas life satisfaction focuses more directly on subjective responses to those conditions and on the appraisal of life as a whole (7). The two variables were strongly correlated in this study (*r* = 0.70), but they need not always move in the same direction. For example, favorable objective conditions such as economic security, good health, and broad social networks may coexist with lower life satisfaction if one lacks meaning in life. Conversely, even under illness or financial hardship, life satisfaction may remain high if one finds meaning, feels gratitude, or copes effectively with such adversity. This distinction supports the decision to examine both outcomes.

The distinction also has policy relevance. Policy often aims not only to improve objective living conditions but also to improve how people evaluate their lives. Quality of life and life satisfaction may influence each other, but the conceptual and empirical gap between them suggests that interventions should not assume that improving one automatically improves the other. In this context, the finding that resilience partially mediated the associations of loneliness with both outcomes is meaningful. If future intervention studies confirm these pathways, strengthening resilience may provide a strategy relevant to both quality of life and life satisfaction, which may be useful given the challenges of separately intervening on each of the two outcome variables.

The measurement of quality of life and life satisfaction in this study was based on a single item and a recategorized single item, respectively, and the findings should therefore be interpreted cautiously. Single-item measures reduce respondent burden in large surveys and can capture global evaluations. However, they may not fully represent the subdomains of quality of life or the psychometric structure of life satisfaction. Future studies should use validated multi-item measures of quality of life and life satisfaction to assess these constructs more comprehensively.

The findings of this study provide several implications for community-based mental health and wellbeing programs for older adults. First, loneliness and social disconnectedness should be screened at an early stage in community service settings such as senior welfare centers, public health centers, home-visiting health care services, and community mental health welfare centers. Because loneliness is not the same as living alone, screening should assess subjective relationship satisfaction and emotional connectedness, rather than merely checking whether an older adult lives with family members or how often they have social contact.

Second, although the negative associations of loneliness with quality of life and life satisfaction call for policy responses, direct intervention to reduce loneliness can be difficult. One reason is that loneliness is often a consequence rather than a cause. For instance, if a person feels lonely due to having few close associates, providing such associates cannot be a viable option. Intervention may also be more difficult when loneliness reflects long-standing interpersonal patterns or stable tendencies in how individuals perceive and interpret social relationships. The causes of loneliness also vary substantially across individuals, further compounding the difficulty. Another reason is that loneliness is an emotional state, and emotional experience itself is not as directly trainable as coping skills or psychological resources.

By contrast, resilience is a psychological and coping resource rather than an emotion itself. It includes skill-based components that may be strengthened through training and through experience of coping with adversity. Thus, resilience may offer an additional policy option that is also a more feasible point for intervention and practice. Approaches to strengthen resilience in later life may help improve psychological resources, and life review or reminiscence therapy, positive psychology-based activities, problem-solving training, and group counseling may be considered as potential strategies (25, 26).

Third, resilience-building efforts could be considered alongside efforts to strengthen social connection. The partial mediation patterns indicate that intrapersonal psychological resources alone do not fully account for the association between loneliness and lower wellbeing. Interventions for loneliness in later life may therefore benefit from extending beyond individual counseling or education and from incorporating expanded opportunities for community participation, peer network formation, improved digital access, and stronger social support systems. This is consistent with prior meta-analytic evidence that loneliness-reduction interventions may include social skills training, increased opportunities for social contact, strengthened social support, and modification of maladaptive social cognition (27). A meta-analysis of interventions for loneliness and social isolation among older adults also reported that exercise, technology-based interventions, psychotherapy, and multicomponent interventions may be associated with reduced loneliness (28). Such an integrated approach is also consistent with international discussions emphasizing social connection as a core element of health and wellbeing (3).

This study has several strengths. First, it used data from a nationwide mental health survey to examine associations among loneliness, resilience, quality of life, and life satisfaction in Korean older adults. Second, it analyzed two positive wellbeing indicators, quality of life and life satisfaction, and showed that the associations involving loneliness and resilience were consistent across both outcomes. Third, unlike prior studies using the same data that focused mainly on mental disorders or negative mental health outcomes, this study examined wellbeing in later life with resilience as a potentially modifiable psychological resource. Fourth, by showing partial mediation for both outcome variables, the study suggests a multilevel direction for future intervention research that considers both intrapersonal psychological resources and social connectedness.

Several limitations should also be noted. First, because the study used cross-sectional data, temporal ordering and causal relationships among loneliness, resilience, quality of life, and life satisfaction cannot be established. The mediation results should therefore be interpreted as statistical mediation patterns or structural associations, rather than as causal mediation. Second, the loneliness-related measure used in this study is a brief scale that includes items on loneliness, social support, and social networks, and its internal consistency in this sample was modest (Cronbach’s alpha = 0.67). The results may therefore reflect the composite associations of loneliness and social disconnectedness rather than a unidimensional loneliness construct alone. Third, quality of life and life satisfaction were measured using a single item or a recategorized single item, which limits psychometric precision. Fourth, because the NMHSK included respondents aged 18–79 years, the older-adult subsample did not include adults aged 80 years or older. Fifth, mediation was tested using the Baron and Kenny approach and the Sobel test. The Sobel test assumes a normal approximation to the sampling distribution of the indirect effect. The present sample size (*n* = 1,037) provides some support for this approximation, but future studies should use bootstrap-based confidence intervals or structural equation modeling to estimate the magnitude and uncertainty of indirect effects more precisely. Sixth, although resilience was interpreted as a potentially modifiable psychological resource, this study was not an intervention study. It therefore cannot show that strengthening resilience would improve quality of life or life satisfaction.

Future studies should use longitudinal data to examine the temporal ordering among loneliness, resilience, quality of life, and life satisfaction. They should also distinguish loneliness from social isolation and include objective social networks, subjective relationship satisfaction, social support, depressive and anxiety symptoms, physical health, economic vulnerability, and community environmental factors in multivariable models. Randomized controlled trials or quasi-experimental studies are needed to determine whether resilience-building programs improve quality of life and life satisfaction among older adults who experience loneliness. Future research should also measure quality of life and life satisfaction using multi-item scales and examine whether mediation pathways differ by sex, age, living arrangement, and economic vulnerability. Such work could inform more targeted community-based interventions.

## 5 Conclusion

This study showed that, among Korean older adults, loneliness was associated with lower quality of life and lower life satisfaction, and that resilience may partly account for these associations. The study contributes to the literature by using nationwide mental health survey data to examine loneliness, resilience, quality of life, and life satisfaction together, and by showing a consistent partial mediation pattern across two positive wellbeing indicators. The partial mediation pattern suggests that the association between loneliness and lower wellbeing cannot be explained by intrapersonal psychological resources alone. Social support and networks, physical health, economic conditions, and community environments should also be considered.

From a policy perspective, this study offers two implications. First, because loneliness in later life was associated with both quality of life and life satisfaction, early screening and preventive responses may be warranted in community service settings. Second, although the emotion of loneliness itself is difficult to control directly, resilience may be a more feasible intervention point because it reflects psychological resources and coping capacity. Future intervention research could therefore evaluate programs that combine resilience-building with community-based approaches to reduce loneliness and strengthen social connection. Because this study was based on cross-sectional data and brief survey items, causal interpretations should be made cautiously. Future research should use longitudinal designs, refined psychometric instruments, bootstrap-based mediation testing, and intervention studies to clarify the psychological pathways linking loneliness and wellbeing in later life.

## Data Availability

The original contributions presented in the study are included in the article/supplementary material, further inquiries can be directed to the corresponding author.
